# Delayed respiratory syncytial virus epidemic in children after relaxation of COVID-19 physical distancing measures, Ashdod, Israel, 2021

**DOI:** 10.2807/1560-7917.ES.2021.26.29.2100706

**Published:** 2021-07-22

**Authors:** Moran Weinberger Opek, Yonatan Yeshayahu, Aharona Glatman-Freedman, Zalman Kaufman, Nadav Sorek, Tal Brosh-Nissimov

**Affiliations:** 1 Department of Pediatrics, Samson Assuta Ashdod University Hospital, Ashdod, Israel; 2 Faculty of Health Sciences, Ben-Gurion University in the Negev, Beer-Sheva, Israel; 3 Israel Center for Disease Control, Ministry of Health, Tel Hashomer, Ramat Gan, Israel; 4 Department of Epidemiology and Preventive Medicine, School for Public Health, Sackler Faculty of Medicine, Tel Aviv University, Tel Aviv, Israel; 5 Microbiology laboratory, Samson Assuta Ashdod University Hospital, Ashdod, Israel; 6 Infectious Diseases Unit, Samson Assuta Ashdod University Hospital, Ashdod, Israel

**Keywords:** respiratory syncytial virus (RSV), seasonality, summer, COVID-19, physical distancing, palivizumab administration

## Abstract

Following low incidence of respiratory syncytial virus (RSV) infections in 2020 during the COVID-19 pandemic, we noted a resurgence in hospitalised children in spring/summer 2021 following relaxation of public health measures. We compared this outbreak to previous autumn/winter seasons. We found higher weekly case numbers and incidence rates, more cases from urban neighbourhoods with lower socioeconomic status, and similar clinical presentation and severity. Public health implications include the re-evaluation of palivizumab administration and the need for surge capacity planning.

An unprecedented low incidence of respiratory viral infections was reported during the coronavirus disease 2019 (COVID-19) pandemic [1-6]. The likely contributing factors included universal mask use, school closures, travel restrictions, bans on mass gatherings and other public health and physical distancing measures to control COVID-19. Following the relaxation of COVID-19 measures, an out-of-season increase in respiratory viral infections, including respiratory syncytial virus (RSV), was observed in Israel [7].

We noticed a resurgence in RSV infection in paediatric inpatients in our hospital since May 2021. We describe this uncommon spring/summer RSV outbreak by comparing it to previous autumn/winter seasons.

## Shift in respiratory syncytial virus seasonality after relaxation of COVID-19 measures

Assuta Ashdod is a University hospital with 300 beds that started its operation in November 2017 and provides almost all acute inpatient care to a city of ca 220,000 people. Paediatric inpatients with acute respiratory infections are routinely tested for RSV and influenza using the express FLU kit (GeneXpert system, Madison, Sunnyvale, CA, United States), or the FLU/RSV direct kit (Liason MDX, Diasorin, Saluggia, Italy). Tests for other respiratory viruses are performed for patients admitted to the intensive care unit (ICU) or at the request of attending paediatricians.

We analysed RSV test data from hospitalised paediatric patients between 1 January 2018 and 24 June 2021. The weekly incidence was calculated by dividing the number of cases by the age-specific population in Ashdod. The incidence was calculated separately for each year, using an updated census reported by the municipality.

We extracted data for all 70 RSV cases in spring/summer (May−June) 2021 and for 140 RSV-positive patients from 2018 to 2020, chosen by a computerised randomisation algorithm. Demographic and clinical data (e.g. comorbidities, clinical manifestations, coinfections, length of stay in hospital, treatment during hospital stay, and ICU admissions) were retrieved from patients' medical records. Patients' addresses were used to assign a geostatistical area (GSA) with a defined average socioeconomic status (SES) to each case in order to provide an average socioeconomic score. Comparisons between cases and controls were accomplished using the Fisher's exact and Mann–Whitney tests, using GraphPad Prism version 9.1.2.

Between 1 January 2018 and 24 June 2021, 5,263 RSV tests were performed, and 430 (8%) of those were positive. The weekly number of cases and annual peaks are presented in Figure. The weekly incidence rate followed the same pattern and peaked at 34 per 100,000 and 38 per 100,000 children during the autumn/winter season in 2018/19 and 2019/20 respectively, and at 50 per 100,000 during the spring/summer of 2021.

**Figure:**
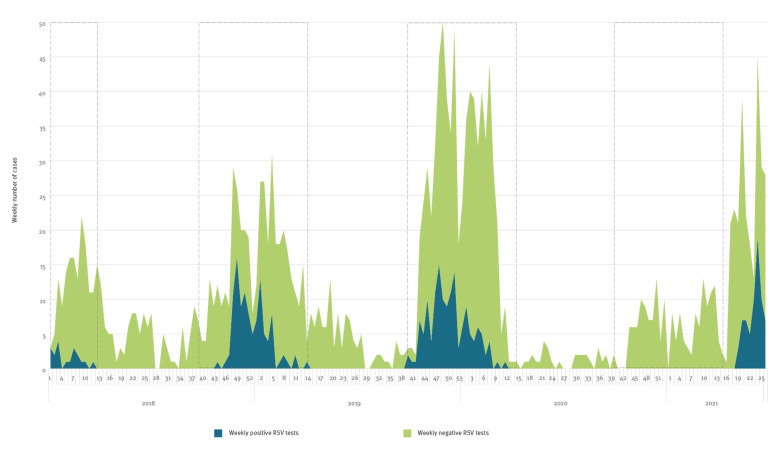
Weekly number of positive and negative respiratory syncytial virus tests, 1 January 2018−24 June 2021

RSV: respiratory syncytial virus.

Weekly positive RSV tests (blue area), with negative tests stacked on top (green area). The dashed rectangles mark the autumn/winter RSV seasons (calendar weeks 40–14). During 2021, no positive RSV cases were noted in autumn/winter but have appeared since week 19.

Between 2018 and 2020, all RSV cases were detected between week 40 and week 14. By comparison, of the 172 RSV tests performed during the autumn/winter of 2020, none were positive. Between 1 May 2021 and 24 June 2021 (weeks 19–26), 70 RSV cases were detected from 430 patients tested (16%).

## Comparison of the summer/spring 2021 epidemic with previous autumn/winter seasons

The 70 RSV cases during spring/summer 2021 were compared with 140 random cases for comparison from 2018 to 2020 (Table). The representation of each comparison season was similar in the randomly selected cohort, with 43/108 (43%), 65/175 (37%) and 32/79 (41%) cases selected from the 2017/18, 2018/19 and 2019/20 RSV seasons, respectively (Fisher's exact test, p = 0.84). Sex was found to be different compared with previous seasons, with more males than females in earlier seasons (p = 0.04), and an equal distribution in spring/summer 2021. More cases in the spring/summer 2021 epidemic lived in the city of Ashdod as opposed to suburbs than in previous autumn/winter seasons (80% vs 59%, p < 0.01) and resided in areas with a lower SES (p < 0.0001). Other characteristics, including age, comorbidities, clinical presentations, disease severity, treatments, and length of stay in hospital were similar (Supplementary table S1).

**Table:** Comparison of demographic characteristics between RSV cases during spring/summer 2021 and a random sample of RSV cases from autumn/winter seasons 2018/19 and 2019/20, Ashdod, Israel

		Randomly selected RSV cases from 2018/19 and 2019/20		RSV cases spring/summer 2021		p value
		n	%	n	%	
N		140	NA	70	NA	NA
Admission during routine palivizumab administration period^a^		130	93	0	0	**< 0.0001**
Age: median (IQR), months		7 (3–14)	NA	6 (2–14.3)	NA	> 0.99
Sex	Male	92	66	35	50	**0.04**
	Female	48	44	35	50	
Municipality	Ashdod	83	59	56	80	**< 0.01**
Average socioeconomic score (SES) in GSA^b^	2	4	NA	11	NA	**< 0.0001**
	3	35	NA	36	NA	
	4	14	NA	7	NA	
	5	21	NA	6	NA	
	6	29	NA	6	NA	
	7	22	NA	3	NA	
	8	5	NA	1	NA	
	9	1	NA	0	NA	

GSA: geographic statistical area; IQR: interquartile range; SES: socioeconomic score; RSV: respiratory syncytial virus.

^a^ Routine palivizumab administration period: November–March.

^b^ Socioeconomic score – a higher rank (between 1 and 9) signifies a higher socioeconomic score.

### Ethical statement

The study was approved by the Assuta Ashdod ethical board (RN 0096–21-AAA). Informed consent was waived given the retrospective nature of the study. Analysis was performed on de-identified data.

## Discussion

Our study showed a temporal shift of the RSV epidemic during 2021 among hospitalised children in Ashod, with complete absence of cases during the autumn/winter of 2020/21, and a resurgence of cases in May to June 2021. The 2021 spring/summer epidemic exceeded previous autumn/winter seasons in terms of weekly case counts and incidence in our paediatric population. Compared with previous seasons, the 2021 spring/summer epidemic included more children from the city of Ashdod as opposed to suburbs, and mainly from neighbourhoods with lower SES. This pattern suggests higher incidence in more densely populated areas but might also represent earlier relaxation of COVID-19-related physical distancing measures in these populations. We did not find differences in age, comorbidities, clinical presentation, or disease severity.

The Israeli COVID-19 vaccination campaign began in December 2020 with ensuing decline of COVID-19 incidence, and a notable impact on transmission [8]. Gradual relaxation of public health measures such as physical distancing began in February 2021 and proceeded with school reopening, permission of gatherings, and cessation of mandatory mask use. The RSV incidence rate notably increased following the relaxation of these measures. Nevertheless, the 2021 out-of-season RSV epidemic is surprising. Attempting to explain the autumn/winter seasonality of respiratory viral infections in temperate areas, climatic factors such as temperature, humidity and ultraviolet radiation have been implicated as contributors [9-11]. Another study on an RSV outbreak in Minnesota in the summer of 2021 linked to a novel lineage illustrated the importance of population immunity for RSV seasonality [12]. Our study further emphasises this, as RSV autumn/winter season-like incidence rates appeared during the spring/summer of 2021 in a susceptible population. Most spring/summer cases (75%) of 2021 were born at the beginning of March 2020, when RSV incidence rates decreased to zero. Therefore, they reached the spring/summer of 2021 with no previous exposure or development of immunity. Furthermore, these children were born to mothers with no RSV exposure during pregnancy, and since neutralising antibodies to RSV are short-lived [13], no significant transfer of RSV-specific antibodies could have been expected.

The shift in RSV seasonality reflects similar findings reported elsewhere. In Australia a 98.0% and 99.4% reduction in RSV and influenza detection were shown, respectively, throughout the southern hemisphere autumn/winter season (March−September) of 2020 [5], followed by an RSV surge as physical distancing restrictions were relaxed [14]. An increase in RSV cases and patients' median age was attributed to an expanded cohort of RSV-naïve patients. Likewise, a recent study from the United States (US) has shown no reported RSV cases from September 2020 to January 2021, followed by an increase from February through to May 2021 [15]. The study also showed a more severe disease course, explained by diminished immunity from lack of previous exposure. In contrast, we did not find differences in disease severity. These two articles and our report reflect a trend reported by surveillance systems including the system set up by the US Centres for Disease Control and Prevention (CDC), with a 60-fold increase in RSV case count (1,260 vs 21) in week 28 of 2021 compared with the same week of the previous year [16]. We therefore consider our findings as portending a global phenomenon in conjunction with relaxation of physical distancing measures.

In contrast to the previous studies, our results did not show differences in the affected children’s age. It is possible that this epidemic also involved older children in the community, but our hospital-based cohort only reflects cases severe enough to be admitted, which are generally comprised of infants and younger children. Our data shows an equal sex distribution in spring/summer 2021, contrasting with the 1.5-fold male predominance observed in previous years. In a national-wide analysis in Israel between 2000 and 2017, a ca 1.4-fold male predominance in hospitalised paediatric RSV cases was found [17]. Indeed, males are at an increased risk for paediatric respiratory morbidity, mainly bronchiolitis, possibly due to foetal-related factors influencing lung development [18]. Our findings may reflect unknown differences in immunity between the sexes, or behavioural differences after the relaxation of measures, such as changes in the segregation of sexes in educational facilities commonly practiced by religious communities in our city, but might also be attributed to our relatively small cohort of spring/summer 2021.

In light of our findings, some public health implications should be emphasised. Serious morbidity because of RSV in premature children and children with pulmonary comorbidities can be prevented by palivizumab, a monoclonal antibody against RSV. Five doses are recommended during the autumn/winter season (November–March) in many countries in the northern hemisphere [19]. Most children (130/140, 93%) with RSV during the 2018/19 and 2019/20 seasons were admitted to Assuta Ashdod Hospital within this period, in contrast with no such admissions during 2020/21. This points to a need for a re-evaluation of palivizumab administration by respective national authorities in countries with similar delays in their local epidemic, as is being presently done by the Israeli Ministry of Health. Another implication of a seasonal shift is its potential impact on medical services. Countries relaxing their pandemic countermeasures may need to plan a consequent capacity surge considering groups at risk for RSV. This might also be important for future pandemic planning.

This study has several limitations. Its retrospective nature, implies that there was potential for ascertainment bias. However, our institutional testing strategies did not change between past seasons and the current outbreak and included RSV testing for every patient with an acute respiratory infection. A relatively small sample size for the 2021 cases might have prevented an accurate comparison for risk factors and outcomes. Moreover, our hospital started operating in November 2017, and therefore, only three previous autumn/winter seasons were available for comparison. Being single-centred, our study might not represent other communities in Israel. We did not perform any typing for the RSV isolates, and we cannot comment on the antigenic similarity of this epidemic to previous ones. Finally, we report early findings (i.e. before this year’s RSV epidemic has ended), and therefore cannot predict the magnitude or length of the RSV surge. Yet, these timely findings might have an impact on public health and may influence future control measures.

## Supplementary Data

234000 Supplementary Material
