# Supplementary material for: Delayed respiratory syncytial virus epidemic in children after relaxation of COVID-19 physical distancing measures, Ashdod, Israel, 2021
Source: Euro Surveill. 2021 Jul 22;26(29):2100706. doi: 10.2807/1560-7917.ES.2021.26.29.2100706 (PMC8299746; doi:10.2807/1560-7917.ES.2021.26.29.2100706)
Supplement: Supplementary Material [file 21-00706_WEINBERGER-OPEK_SupplementaryTableS1.pdf]

This supplementary material is hosted by *Eurosurveillance* as supporting information alongside the article "Delayed respiratory syncytial virus season in spring-summer after relaxation of COVID-19 social distancing restriction in Ashdod, Israel, 2021", on behalf of the authors, who remain responsible for the accuracy and appropriateness of the content. The same standards for ethics, copyright, attributions and permissions as for the article apply. Supplements are not edited by *Eurosurveillance* and the journal is not responsible for the maintenance of any links or email addresses provided therein.

S1: Comparison of clinical characteristics between RSV cases of spring-summer 2021 and a random sample of comparison cases from autumn-winter seasons 2018-2020.

		**2018-2020 comparison**		**2021**		**P value**
		**n**	**%**	**n**	**%**	
N		140		70		
Comorbidities	Recurrent wheezing	32	23	16	23	>0.99
	Congenital heart diseases	4	3	2	3	>0.99
	Atopic dermatitis	6	4	3	4	1.0
	Bronchopulmonary dysplasia	2	1	0	0	0.55
	Neurological or muscular diseases	7	5	0	0	0.1
Prematurity	Any	17	12	4	6	0.22
	33-36 w	14	10	4	6	
	29-32 w	3	2	0	0	
	≤28 w	0	0	0	0	
Length of stay - median (IQR),days		3 (2-5)		3 (1-6)		0.39
ICU admission	Overall	31	22	21	30	0.24
	Length of stay - median (IQR), days	3 (2-4)		3 (2.5-5)		0.32
Clinical manifestations	Fever	117	84	52	74	0.14
	Cough	128	91	68	97	0.15
	Tachypnea±dyspnea	110	79	57	81	0.72
Respiratory support	Oxygen supplementation	71	51	38	54	0.66
	HFNC	30	21	20	29	0.3
	Mechanical ventilation	6	4	2	3	0.72
Intravenous hydration		94	67	44	63	0.54
Antimicrobial treatment		88	63	39	56	0.37
Coinfection with other viruses	Positive PCR - N/number tested	11/38	29	9/23	45	0.57
	Adenovirus	4/38	10	5/23	22	
	Enterovirus	2/38	5	2/23	9	
	hMPV	0/38	0	1	4	
	PI3	1/38	3	1	4	
	Influenza A	1/38	3	0	0	

Abbreviations: IQR, interquartile range; ICU, intensive care unit; HFNC, high flow nasal canula support; PCR, polymerase chain reaction; hMPV, human metapneumovirus; PI3, parainfluenza virus 3.
